# Editorial: Knowledge graph technologies: the next Frontier of the food, agriculture, and water domains

**DOI:** 10.3389/frai.2023.1319844

**Published:** 2023-11-28

**Authors:** Catherine Roussey, Christophe Guéret, Marie-Angélique Laporte

**Affiliations:** ^1^MISTEA, INRAE & Institut Agro, University of Montpellier, Montpellier, France; ^2^Accenture, Technology Innovation Labs, The Dock, Dublin, Ireland; ^3^Digital Inclusion, Bioversity International, Montpellier, France

**Keywords:** agriculture, food, knowledge graph, ontology, graph database, Semantic Web, property graph, inference

A Knowledge Graph (KG) is based on a graph model to encode the description of entities. As defined by Hogan and his collaborators in 2022, a knowledge graph is “a graph of data intended to accumulate and convey knowledge of the real world, whose nodes represent entities of interest and whose edges represent relations between these entities.” For Knowledge Graph using Semantic Web technologies, entities (people, events, concepts, etc.) are identified by a Uniform Resource Identifier (URI). This URI is the source of a graph description, the edge specifies the nature of the link (person name or brotherhood relationship) and the destination of the edge could be a simple literal (the person name) or a URI that identifies another entity (the URI of the brother). The main advantage of these technologies is to link entities that are described differently in several knowledge graphs provided by various organizations. Thus, computer scientists may analyze all those graph descriptions to derive new information (detect incoherencies, complete data, etc.).

During the last decade, considerable progress has been made in the construction and enrichment of KGs, including ontology matching, data integration, fact prediction, and validation. This happened largely thanks to the use of techniques developed in the fields of knowledge representation, reasoning, and machine learning. With these advances, more and more applications are now able to produce and process KGs in domains such as life sciences, Galleries/Libraries/Archives/Museums (GLAMs), and health care. The subjects of interest within the Food, Agriculture, and Water domains are often complex phenomena where entities evolve through time and space. Those phenomena may be transformed by different processes and influenced by both human and natural systems. The scientific disciplines that study these phenomena are diverse and do not necessarily share the same vocabularies, the same techniques of observation, the same analyses, and so on. Indeed, each discipline often has its own point of view to describe the complexity of the studied phenomena. KG technologies provide one possible approach to express this diversity of representations and align or combine them.

This Research Topic has received 13 abstracts, from which 8 articles were accepted.

Three articles present a method, 4 articles are original research, and 1 is a conceptual analysis. Overall they cover three broad Research Topics often discussed in the KG research communities: ontologies design, data architectures, reasoning.

Ontologies are the back-bone of KG modeling as they define what is in the data and how the information is connected. The Research Topic covers this import topic with three publications:

- “*C3PO: a crop planning and production process ontology and knowledge graph*” by Darnala et al. presents the design method to build and update a modular ontology and associated knowledge graph about vegetable production and planification activities. Some new design patterns are defined dedicated to agriculture. For example, the set of planned tasks that compose a technical itinerary of a crop type are presented. The final C3PO knowledge graph was used by the Elzeard enterprise to build three decision information systems.

- “*EPPO ontology: a semantic-driven approach for plant and pest codes representation*” by Ayllón-Benitez et al. presents the translation of European and Mediterranean Plant Protection Organization (EPPO) database into an OWL ontology. Each entity identified by an EPPO code becomes an OWL class. The ontology will be used as lingua franca to search data into different information systems used in BASF.
- “*Ontological how and why: action and objective of planned processes in the food domain*” by Dooley and Naravane present an extension of the FoodOn ontology about food processes. They propose two new types of process representations: processes by objectives, processes by mechanisms. Their goals are to improve search capability and identification.

An ontology on its own is not much use without data to instantiate it. The past decades of research into KG saw several approaches being presented to combine and align different data into a KG. Not all of those apply straight away to the agricultural domain and this Research Topic features 4 articles proposing specialized innovative approaches:

- “*CowMesh: a data-mesh architecture to unify dairy industry data for prediction and monitoring*” by Pakrashi et al. presents an approach to integrate data in the dairy industry by leveraging a combination of data mesh and data fabric design pattern. The approach is presented from a general point of view along with two specific use-case examples for the dairy industry.
- “*Development of a knowledge graph framework to ease and empower translational approaches in plant research: a use-case study on grain legumes*” by Imbert et al. presents the design method of a Neo4J graph database that integrates the trait and gene information extracted from several sources. The graph model reuses existing ontologies like the Gene Ontology (GO), the Plant Ontology (PO) and the Plant Experimental Condition Ontology (PECO). The method was applied on the database design related to five legume species.
- “*Combining different points of view on plant descriptions: mapping agricultural plant roles and biological taxa*” by Amardeilh et al. presents some guidelines to publish a mapping dataset between two knowledge graphs: The French Crop Usage thesaurus defined crop usage expressed in French. TAXREF is the nomenclatural and taxonomic repository of living organisms that appear in French territories. A new specialized RDF vocabulary of mapping is defined and presented.
- “*Integrating collective know-how for multicriteria decision support in agrifood chains—application to cheesemaking*” by Buche et al. presents a multi-criteria decision support system (MDCSS) based on the capture and modelization of collective know-how in a Knowledge Graph. The ontology for expressing this information is introduced together with an example application for the process of cheese making.

Lastly, to illustrate the “Knowledge” part of a KG and reasoning over this knowledge, we have in this issue one paper covering using a KG to infer new information:

- “*Using knowledge graphs to infer gene expression in plants*” by Thessen et al. illustrates how a knowledge graph connecting partial information available about different plants can lead to new insights. Leveraging homologous genes as an inference back-end it is possible, as shown, to infer some of the unknown phenotypic impacts of plants gene regulatory networks.

We would like to thank the authors who submitted articles, the reviewers who evaluated them and the external editors who managed the reviews. All these people helped build a quality program for this Research Topic.

## Author contributions

CR: Writing—original draft, Writing—review & editing. CG: Writing—original draft, Writing—review & editing. M-AL: Writing—original draft, Writing—review & editing.

